# Sister chromatid telomere fusions, but not NHEJ-mediated inter-chromosomal telomere fusions, occur independently of DNA ligases 3 and 4

**DOI:** 10.1101/gr.200840.115

**Published:** 2016-05

**Authors:** Kate Liddiard, Brian Ruis, Taylor Takasugi, Adam Harvey, Kevin E. Ashelford, Eric A. Hendrickson, Duncan M. Baird

**Affiliations:** 1 Institute of Cancer and Genetics, School of Medicine, Cardiff University, Heath Park, Cardiff, CF14 4XN, United Kingdom;; 2 Department of Biochemistry, Molecular Biology, and Biophysics, University of Minnesota Medical School, Minneapolis, Minnesota 55455, USA

## Abstract

Telomeres shorten with each cell division and can ultimately become substrates for nonhomologous end-joining repair, leading to large-scale genomic rearrangements of the kind frequently observed in human cancers. We have characterized more than 1400 telomere fusion events at the single-molecule level, using a combination of high-throughput sequence analysis together with experimentally induced telomeric double-stranded DNA breaks. We show that a single chromosomal dysfunctional telomere can fuse with diverse nontelomeric genomic loci, even in the presence of an otherwise stable genome, and that fusion predominates in coding regions. Fusion frequency was markedly increased in the absence of TP53 checkpoint control and significantly modulated by the cellular capacity for classical, versus alternative, nonhomologous end joining (NHEJ). We observed a striking reduction in inter-chromosomal fusion events in cells lacking DNA ligase 4, in contrast to a remarkably consistent profile of intra-chromosomal fusion in the context of multiple genetic knockouts, including DNA ligase 3 and 4 double-knockouts. We reveal distinct mutational signatures associated with classical NHEJ-mediated inter-chromosomal, as opposed to alternative NHEJ-mediated intra-chromosomal, telomere fusions and evidence for an unanticipated sufficiency of DNA ligase 1 for these intra-chromosomal events. Our findings have implications for mechanisms driving cancer genome evolution.

Short telomeres inadequately protected by the shelterin complex (de Lange 2005) are recognized as double-stranded DNA breaks (DSBs), activating nonhomologous end-joining (NHEJ) DNA repair that can result in chromosomal fusions (Cowell and Miller 1983; Counter et al. 1992; Capper et al. 2007). Two fundamentally distinct pathways of NHEJ have been characterized in eukaryotes based on the differential usage of DNA ligase 4 (LIG4; classical) or DNA ligase 3 (LIG3; alternative) (Boboila et al. 2012; Oh et al. 2014). The rapid kinetics and high avidity of Ku complex binding to initiate classical NHEJ (C-NHEJ) repair precludes extensive processing of the DNA lesion (Shibata et al. 2011). In contrast, the alternative NHEJ (A-NHEJ) pathway involving both LIG3 and poly(ADP-ribose) polymerase 1 (PARP1) (Pascal and Ellenberger 2015) is associated with greater substrate resection to reveal microhomology that bridges and stabilizes junctions. More recently, DNA ligase 1 (LIG1) has been implicated as a functional substitute for LIG3 in chicken cells (Arakawa et al. 2012) and a subset of LIG3 activities in mouse cells (Lu et al. 2016). The interdependence or hierarchy of specialized LIG1 and LIG3 roles in A-NHEJ remains to be clarified in human cells. We have previously documented the essential role of LIG3 in the fusion of critically short telomeres capping sister chromatids in cells undergoing a telomere-driven crisis (Jones et al. 2014). We consider that such intra-chromosomal telomere fusions may endow cells with the capacity for escape and recovery of mitotic function following localized sequence amplifications or deletions (Murnane 2010) that ultimately up-regulate telomerase. Conversely, telomere fusions mediated by LIG4 are either insufficient to support transforming genomic rearrangements or are incompatible with cell viability. Mitotic slippage in the absence of cell cycle regulators, including tumor protein p53 (TP53), increases the fusogenic substrate pool and extends the lifespan of genetically unstable cells (Davoli et al. 2010; Hayashi et al. 2015). The nature of the DNA lesions activating different NHEJ pathways (Rai et al. 2010) and the ultimate balance between the resulting long-range inter-chromosomal and short-range intra-chromosomal telomeric fusions significantly impact cellular capacity for evolution and survival.

In this study, we have examined the differential repair of inter- and intra-chromosomal telomere fusion events. The substantial technical challenges associated with characterizing rare and unique telomere fusions (Letsolo et al. 2010) in sufficient number and resolution have historically impeded progress in accurately delineating the mechanisms and consequences of these events (Luo et al. 2011). To specifically redress this, we developed a high-resolution sequencing approach for large-scale molecular profiling of diverse telomere fusion events in human cells with naturally configured telomeres without a priori knowledge of recombination mechanisms or locations. We have used this system to answer salient questions concerning the hierarchy of repair pathways involved in inter-chromosomal and intra-chromosomal telomere fusions, as well as to identify genomic features, including gene content and DNA topology that may inform predictive models of genomic instability.

## Results

### High resolution mapping of inter- and intra-chromosomal recombinations involving eroded telomeres in human fibroblasts

**Figure 1.**
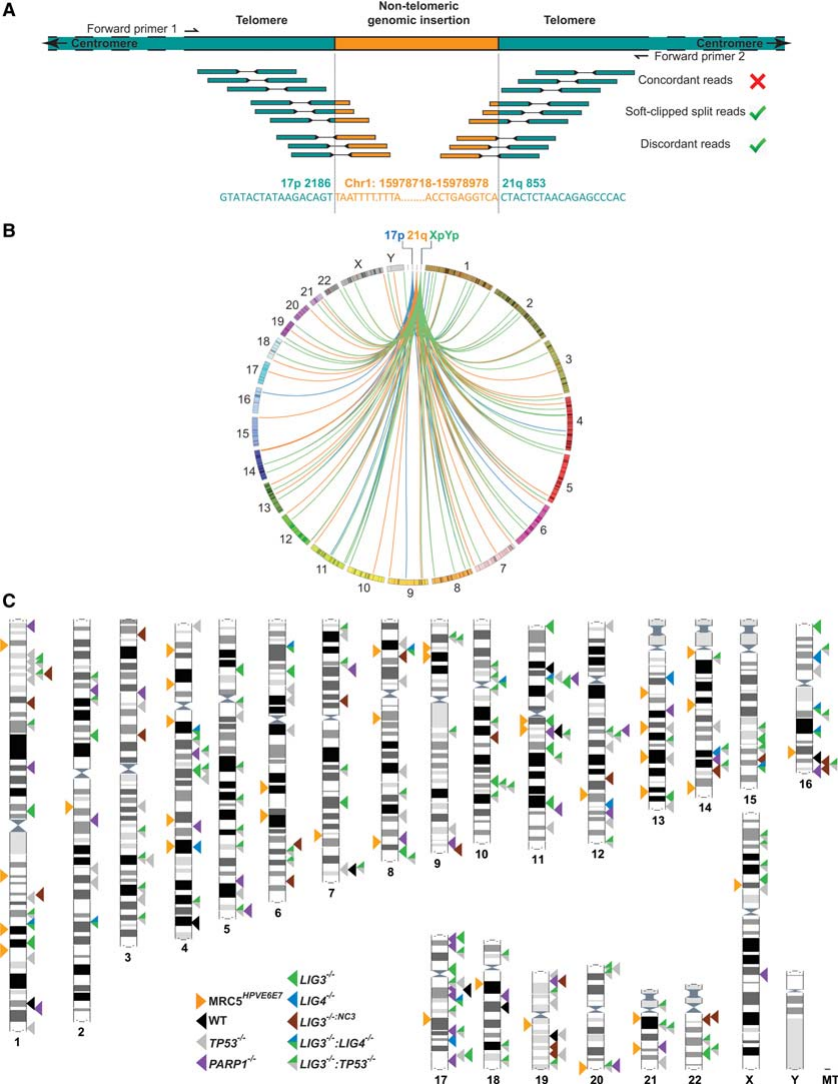
Characterization of telomere-genomic inter-chromosomal fusions in crisis-stage MRC5^*HPVE6E7*^ cells. (*A*) Cartoon representation of an inter-chromosomal fusion between the 17p and 21q telomeres (green) and a genomic locus (orange) amplified from MRC5^*HPVE6E7*^ fibroblasts undergoing telomere-induced crisis and sequenced by Illumina HiSeq 2000 paired-end sequencing. Discordant read pairs are those that do not map to the reference sequence with the expected orientation or size coverage. Soft-clipped reads are those containing mismatches with the reference sequence. Inter-chromosomal telomere-genomic fusion events were defined as those discordant and soft-clipped linkages mapping to at least one telomere end (17p, XpYp, or the 21q family) and a nontelomeric genomic location. Fusion PCR primer orientations are indicated *above* the chromatids. (*B*) Circos plots (Krzywinski et al. 2009) displaying all inter-chromosomal fusion linkages between the 17p, XpYp, and 21q family telomeres and genomic loci sequenced from crisis-stage MRC5^*HPVE6E7*^ cells. A scaled representation of each human chromosome in clockwise orientation with numerical identifiers creates the circumference of the plot. The particular telomeres investigated in this study are featured as separate references at the *top* of the plot. Linkages between the genome and each telomere are distinguished by the color of the lines traversing the plot. (*C*) Karyotype map showing coordinates of all sequence-verified and BLAST-authenticated telomere-genomic inter-chromosomal fusion junctions identified in crisis-stage MRC5^*HPVE6E7*^ cells (arrows on the *left* of the chromosomes) and HCT116 cell lines (arrows on the *right* of the chromosomes) selectively compromised in components of DNA repair pathways and subjected to TALEN-targeted nuclease-induced DSBs at the 17p telomere. Each arrow represents a sequenced fusion junction between a telomere and the genome, and the different samples are distinguished by color, as indicated in the key.

We aimed to determine whether paired-end sequencing of telomere fusion events, amplified at the single-molecule level from human fibroblasts undergoing a telomere-driven crisis, would provide a novel and powerful tool for comprehensive analysis of rare genomic recombination events in the absence of preexistent sequence information. We pooled and sequenced fusion molecule amplicons involving the XpYp and 17p telomeres and the family of homologous telomeres related to the 21q telomere (hereafter referred to as 21q). The fusion amplicons were generated from 400,000 diploid genomes of MRC5^*HPVE6E7*^ primary fibroblast cells at the point of their entry into crisis precipitated by short fusogenic telomeres (Capper et al. 2007; Letsolo et al. 2010). Based on an empirically determined telomere fusion frequency (Letsolo et al. 2010) of 2.13 × 10^−3^/diploid genome, more than 850 unique telomere fusion events were subjected to Illumina paired-end sequencing and mapped to their respective telomeres of origin. We extracted only discordant read pairs indicative of genomic rearrangements (Fig. 1A) for high-resolution analysis of individual telomere-genomic fusion events. We observed a higher abundance of read pairs mapping to genomic linkages with the 21q-homologous telomeres (19,350, compared with 14,380 for XpYp and 5603 for 17p), consistent with the increased targeting potential of the amplification primer for all related 21q family members (Supplemental Fig. 1A; Letsolo et al. 2010). We used haplotype analysis to confirm the involvement solely of the shorter MRC5^*HPVE6E7*^ XpYp allele (Baird et al. 2003) in these sequenced fusions (Supplemental Fig. 1B), consolidating demonstrations of the length-dependent threshold for telomere fusion to occur (Capper et al. 2007; Lin et al. 2014).

We performed manual curation and sequence verification of all telomere linkages exceeding mapping quality (Li 2014) and BLAST alignment (Altschul et al. 1990) score thresholds defined using training data sets (described in the Supplemental Methods). Resultant linkages revealed the participation of multiple telomeres in fusions with diverse genomic locations, indicative of the large-scale de novo genomic instability occurring in our fibroblast crisis model (Fig. 1B,C; Supplemental Fig. 1C). We used soft-clipped read sequence information (Fig. 1A) to confirm precise fusion junction sequences for 70 telomere-genomic linkages (Supplemental Table 1), highlighting the suitability of this bespoke sequencing pipeline for high-resolution simultaneous detection of multiple unique telomere fusion events present at the single-molecule level. These data clearly demonstrate that short dysfunctional telomeres in cells undergoing a telomere-driven crisis are prone to fusion with nontelomeric loci at diverse genomic locations, potentially transmitting replication stress and driving further DSBs and fusion events genome-wide.

### Nucleases targeted to subtelomeric regions initiate telomere fusion events, providing a tractable model of telomere dysfunction and recombination

**Figure 2.**
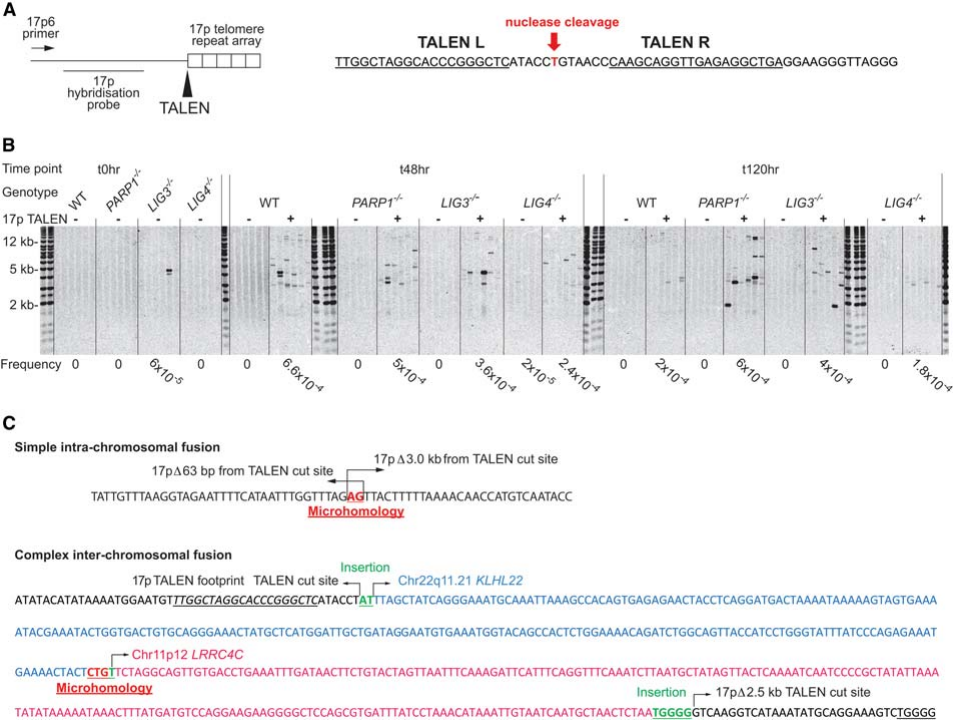
Development of targeted nucleases to induce DSBs and fusions at the 17p telomere. (*A*, *left*) Schematic depicting the subtelomeric region of the 17p Chromosome arm with TALEN target site at the start of the telomere repeat arrays indicated (arrow). The 17p6 primer used to amplify and the location of the 17p hybridization probe used to detect 17p fusion events are shown. (*Right*) The specific recognition sequences bound by the *left* (L) and *right* (R) TALEN nucleases to create a functional heterodimer that cleaves the intervening T residue juxtaposing the 17p telomere hexameric TTAGGG repeats. (*B*) Nucleofection of HCT116 cell lines with the 17p TALEN pair (+) resulted in diverse amplifiable 17p telomere fusions that were absent from untransfected cells (−) at 48 and 120 h post-nucleofection. A sequence-characterized 17p amplicon stochastically detected in the pretransfected (t0) HCT116 *LIG3*^−/−^ cells is shown. Telomere fusions were detected following Southern blotting using the 17p hybridization probe. Fusion frequency estimated empirically is recorded *beneath* the panels. (*C*) Examples of simple intra-chromosomal (*upper*) and complex inter-chromosomal (*lower*) 17p TALEN-induced telomere fusions characterized by Sanger sequencing of reamplified and purified fusion amplicons. The specificity of 17p TALEN targeting is indicated by the proximity of fusion junctions to the TALEN cleavage site in one chromatid: (Δ) deletion from this position. The TALEN recognition site is in italics and underlined. Junction insertions and microhomology are marked *above* and *below* the sequence, respectively. The two distinct loci incorporated are separated by color and annotated with their genomic locations.

Our telomere fusion sequencing indicated that even a single chromosome with a short and dysfunctional telomere might be sufficient to drive widespread genomic recombination events in early crisis-stage human fibroblasts. We hypothesized that DSBs within subtelomeric sequences, which have specialized DNA repair capacity (Miller et al. 2011; Muraki et al. 2015), might activate error-prone repair processes resulting in telomere fusion events, even within telomerase-expressing cells with stable-length telomeres in their natural configuration. To test this hypothesis, we designed transcription activator-like effector nuclease (TALEN) pairs to induce a DSB in the 17p subtelomere at a position 14 bp from the start of the telomeric repeat array with no other identifiable genomic targets (Fig. 2A). Single-molecule 17p-specific telomere fusion events in the approximate range of 1 to 20 kb could be amplified and resolved from 17p TALEN-nucleofected HCT116 colorectal cancer cells within 48 h (Fig. 2B), in the frequency range of 1.8 to 6.6 × 10^−4^/diploid genome, reducing in abundance over the following 7 d. There were no notable effects on 17p telomere length in the bulk populations (Supplemental Fig. 1D), although complex inter-chromosomal fusions, as well as 17p intra-chromosomal fusions were induced by TALEN activity (Fig. 2C). Specificity and efficiency of 17p TALEN targeting was evidenced by the conspicuous predominance of 17p-linked sequence reads (compared to XpYp and 21q-linked reads) obtained by paired-end sequencing of telomere fusion amplicons purified from TALEN-nucleofected HCT116 cell lines (Supplemental Fig. 1C), as well as the preponderance of fusion junctions proximal to the TALEN cleavage site (Fig. 2C; Supplemental Fig. 1E). Overall, 37.72% and 11.56% of all the sequence-characterized HCT116 inter- and intra-chromosomal telomere fusion junctions, respectively, were localized within 25 bp of the TALEN cleavage site (Supplemental Fig. 2A). We therefore consider that telomere-specific TALEN-induced DSBs, together with paired-end sequencing (Supplemental Tables 2A,B) of single-molecule telomere fusions, provides a robust system to examine the mechanisms underlying telomere recombinations and how these can drive large-scale genomic rearrangements.

### Fewer inter-chromosomal telomere fusions occur with genomic loci in the absence of LIG4

To determine the relative contribution of C- (Ku- and LIG4-dependent) or A- (PARP1- and LIG3-mediated) NHEJ repair components (Oh et al. 2014) to the telomere fusion events resulting from 17p TALEN activity, we performed parallel nucleofections into HCT116 cell lines deficient for individual (Oh et al. 2013, 2014; B Ruis, T Takasugi, S Oh, EA Hendrickson, in prep.) and combined components (Supplemental Fig. 2B,C) of these repair pathways, as well as the TP53 cell cycle checkpoint regulator (Hartwell 1992). HCT116 cells depleted of mitochondrial LIG3 are nonviable; hence, the mitochondrial isoform was reconstituted in the HCT116 *LIG3*^−/−^ line, and an additional model of supraphysiological nuclear LIG3 (*LIG3*^−/−:*NC3*^) was created through complementation of this line with cDNA encoding the nuclear isoform (Oh et al. 2014).

17p TALEN transfection reduced cell viability of HCT116 lines compromised in both A-NHEJ and C-NHEJ pathway components compared with wild-type (WT) cells (Supplemental Fig. 2D). Physiologically, all lines experienced a 65%–83% reduction in cell number 48 h (t48) post-nucleofection (Supplemental Fig. 2E), consistent with an episode of mitotic arrest and telomere fusion akin to a telomeric crisis-state (Jones et al. 2014). The WT populations recovered most rapidly, with a 10-fold increase in cell number between 48 and 120 h (t120), coincident with a threefold reduction in 17p fusion frequency (Fig. 2B). Cells with a compound LIG3 and LIG4 deficiency (*LIG3*^−/−^:*LIG4*^−/−^) demonstrated the slowest recovery, with only a 3.3-fold increase in cell number from t48 to t120 compared with an eightfold increase over the same time period prior to nucleofection. DNA was harvested for telomere fusion analysis 48 and 120 h post-nucleofection to compare the initial population contraction and recovery phases of the DNA damage and repair processes. Following fusion amplicon paired-end sequencing, we examined linkages representing inter-chromosomal fusions between the telomeres of 17p, XpYp, or the 21q telomere family and nontelomeric regions of the genome (hereafter termed inter-chromosomal fusions) independently from linkages representing 17p–17p intra-chromosomal telomere fusions.

We focused our comparative analyses on 17p linkages since the specificity of 17p TALEN targeting resulted in a minimal contribution of XpYp and 21q linkages to both inter- and intra-chromosomal fusions (Supplemental Fig. 1C). We calculated the frequency of reads mapping all 17p telomere recombinations relative to genomic DNA input (diploid genomes) to objectively compare the total fusogenic capacity of the different HCT116 DNA repair-compromised lines (Supplemental Fig. 2F). This revealed elevated 17p fusion frequencies in samples deficient for TP53, as well as corroborating the depressed frequencies measured in samples lacking LIG4 (Supplemental Fig. 1B). As with the MRC5^*HPVE6E7*^ crisis-stage fusion data sets, reads mapping inter-chromosomal events were twofold to 14-fold more abundant than reads mapping 17p intra-chromosomal events (Supplemental Fig. 2G; Supplemental Table 2B). The frequency of inter-chromosomal fusions determined for the MRC5^*HPVE6E7*^ sample undergoing multilateral telomere erosion was similar to the HCT116 lines lacking TP53 (Supplemental Fig. 2G) and markedly higher than all other HCT116 lines. Since expression of the viral E6E7 proteins in MRC5 cells suppresses TP53 function (Shay et al. 1993; Li et al. 1998), these data reveal the importance of the TP53 checkpoint for exonucleolytic proof-reading (Akyüz et al. 2002) and the suppression of DNA damage accumulation and NHEJ repair at telomeres (Abu-Odeh et al. 2014), as well as the inhibition of cell division of genetically unstable cells (Bunz et al. 1998; Davoli et al. 2010). Our results confirm a critical role of the TP53 checkpoint in restricting the accumulation of widespread genomic mutations resulting from telomere dysfunction (Hartwell 1992; Hayashi et al. 2015).

**Figure 3.**
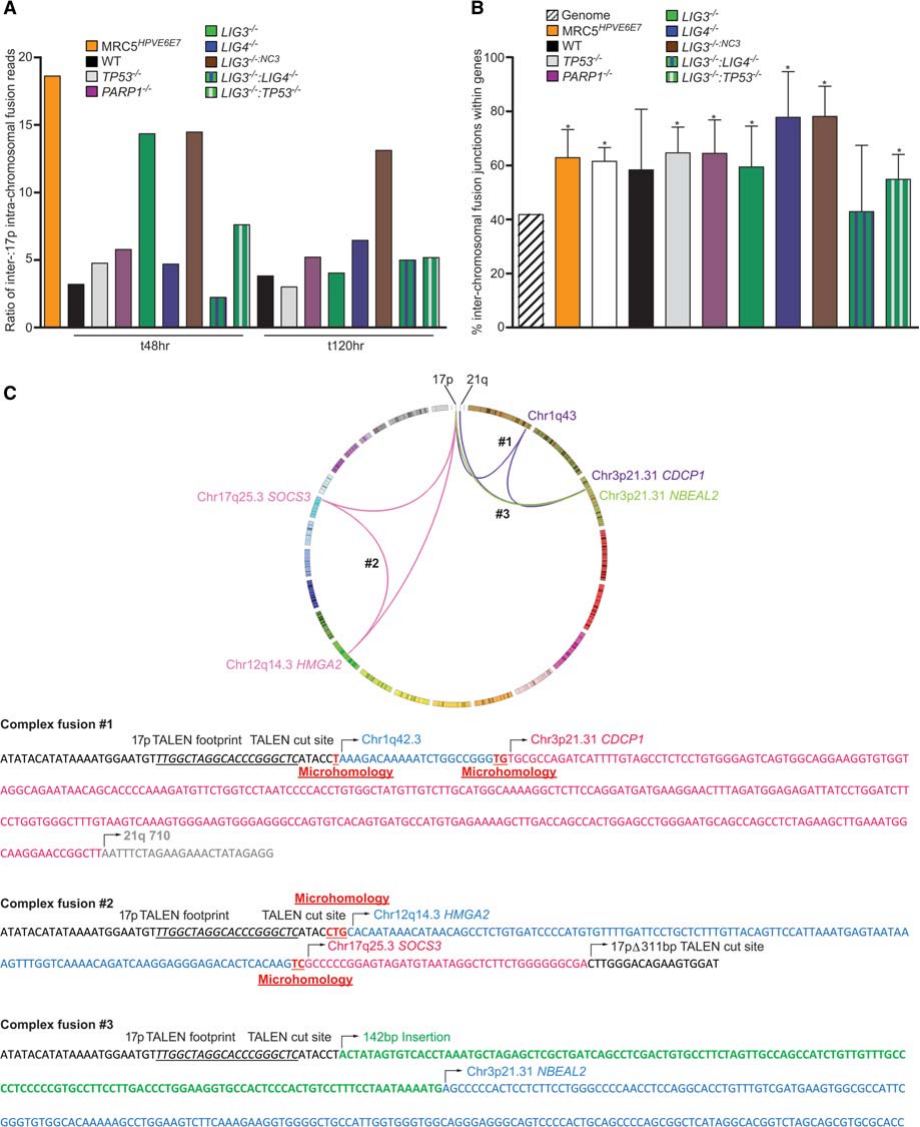
Characteristics of inter-chromosomal fusions between 17p and diverse genomic loci sequenced from MRC5^*HPVE6E7*^ cells undergoing telomere-driven crisis or HCT116 cells undergoing 17p TALEN-induced DNA damage and repair. (*A*) Ratios of Illumina HiSeq 2000 sequence reads mapping to inter-chromosomal telomere-genomic linkages compared with 17p–17p intra-chromosomal linkages are plotted to display enrichment of inter-chromosomal links for all samples. Results relating to specific DNA repair-deficient HCT116 lines are separated by the colors expressed in the key. (*B*) Inter-chromosomal fusion junctions for all samples are more frequently coincident with genes—analysis restricted to Ensembl (Cunningham et al. 2015) and RefSeq (Pruitt et al. 2014) curations—than expected by chance based on RefSeq human genome gene content estimate of 41.8%. Junction locations were investigated using the UCSC Genome Browser (Kent et al. 2002), and statistical significance was determined by χ^2^ analysis for all samples, except HCT116 WT and *LIG3*^−/−^:*LIG4*^−/−^ cells: (*) *P* < 0.05. (*C*) Telomere-genomic fusion events have the potential to be permissive for cellular transformation via telomerase reactivation. A Circos (Krzywinski et al. 2009) plot illustrating three independent inter-chromosomal fusion events between TALEN-targeted 17p and genomic loci implicated in *TERT* regulation—Chr3p21.31 (Cuthbert et al. 1999) and Chr12q14.3;*HMGA2* (Li et al. 2011)—identified by Illumina HiSeq and Sanger sequencing of fusion amplicons from HCT116 *PARP1*^−/−^ (#1 and #2, purple and pink linkages) or *LIG3*^−/−^ (#3, green linkages) cells. The junction sequences of these linkages are presented *below* the Circos plot, with TALEN recognition and cleavage sites, insertions, and microhomology features indicated.

Importantly, based on 17p-linked sequence read numbers, we identified a diametric skew in the ratio of 17p inter-chromosomal:17p intra-chromosomal fusion events sequenced from *LIG4*^−/−^ and *LIG3*^−/−^:*LIG4*^−/−^ cells compared with *LIG3*^−/−^ cells (Fig. 3A). At t48, 17p-linked inter-chromosomal fusion reads were twofold lower in the *LIG4*^−/−^ and *LIG3*^−/−^:*LIG4*^−/−^ compared with the *LIG3*^−/−^ samples (Supplemental Fig. 2G) and the resultant inter-chromosomal:intra-chromosomal ratios were more than threefold lower. This translated into 3.56- and 2.29-fold reductions in sequence-verified inter-chromosomal fusion junctions characterized in *LIG4*^−/−^ and *LIG3*^−/−^:*LIG4*^−/−^, respectively, compared with *LIG3*^−/−^ samples, indicating insufficient compensation for a nonredundant role of LIG4 in these long-range events. We consider that these observations provide evidence for a critical role of LIG4 in effecting inter-chromosomal telomere fusions, rather than indicating an inhibitory impact of LIG3, since we detected comparable increases in inter-chromosomal read frequencies and ratios to intra-chromosomal events for the *LIG3*^−/−:*NC3*^ samples (with supra-normal levels of nuclear LIG3) as with the *LIG3*^−/−^ samples (Fig. 3A). The marked reductions in inter-chromosomal fusion frequency in the absence of LIG4 were mirrored in extra-chromosomal plasmid end-joining assays (Supplemental Fig. 3A–C), independently corroborating the pivotal role of LIG4 in the ligation of divergent substrates with variable requirements for end processing. In contrast, the numbers of 17p intra-chromosomal reads and junctions displayed considerably less variation between samples, even comparing the *LIG3*^−/−^, *LIG4*^−/−^, and *LIG3*^−/−^:*LIG4*^−/−^ samples (Supplemental Fig. 2G). This observation affirms the capacity of all samples for DNA repair responses and reveals the highly context-specific effects of an altered capacity for C-NHEJ compared with A-NHEJ. Combined with the remarkable observation of both inter- and intra-chromosomal telomere fusions in all samples, including *LIG3*^−/−^:*LIG4*^−/−^, this provides important support for the presence of an additional ligase (ostensibly LIG1) (Arakawa et al. 2012; Lu et al. 2016) capable of mediating NHEJ in the absence of LIG3 and LIG4 or an alternative means of functional compensation of this DNA repair pathway in human cells.

### Inter-chromosomal fusions are associated with coding rather than repetitive genomic sequence

Rare telomere-genomic inter-chromosomal fusions have been described in genetically unstable cells, but the low frequency of cases owing to the considerable technical challenges associated with isolating and sequencing these events has made mechanistic analyses impractical (Letsolo et al. 2010; Luo et al. 2011). Our single-molecule approaches to detect telomere fusion in the context of cells undergoing crisis or following telomere-specific DSB induction allowed us to identify a large number of telomere-genomic inter-chromosomal fusion events (Supplemental Table 1). To examine the genomic distributions of these fusions, we plotted all BLAST-validated junction coordinates on a karyotype map (Fig. 1C). Consistent with the initial sequence-read data, the validated junctions clearly demonstrated the relative paucity and contrasting abundance of inter-chromosomal fusion junctions in samples lacking LIG4 (*LIG4*^−/−^, nine events; *LIG3*^−/−^:*LIG4*^−/−^, 13 events) or TP53 (*TP53*^−/−^, 82 events; *LIG3*^−/−^:*TP53*^−/−^, 104 events) (Fig. 1C; Supplemental Fig. 3D), respectively. The fusion junctions were widely dispersed around the genome, but strikingly, they were not correlated with chromosome size, indicating a nonrandom distribution of these loci (Supplemental Fig. 3E). Chromosome 17 exhibited the highest frequency of fusion events per unit length (0.3 junctions/Mb). Fusions involving 17q were identified in all HCT116 lines except the *LIG3*^−/−:*NC3*^, raising the possibility of whole-chromosome instability emanating from TALEN activity at the 17p subtelomere (Fig. 1C). Notably, we detected fusion junctions in nontelomeric regions of 17p in the *PARP1*^−/−^ and *LIG3*^−/−^ samples, although our data did not permit determination of whether these were longer-range intra-chromosomal events or inter-chromosomal events mediated by LIG4. We were able to map fusion junctions over a wide-range of distances from the telomere of each chromosome (Supplemental Fig. 4A), confirming effective capture of long-range inter-chromosomal events.

Contrary to expectation (Stamatoyannopoulos et al. 2009; Supek and Lehner 2015), we discovered a remarkable coincidence of inter-chromosomal fusions with coding sequence (Fig. 3B). Inter-chromosomal fusion junctions for all samples except HCT116 WT and *LIG3*^−/−^:*LIG4*^−/−^ (with their lower frequencies of events) were significantly more likely to occur within genes (overall mean 61.75%; *P* < 0.0001) than would be expected by chance, based on the hg19 RefGene (Pruitt et al. 2014) human genome average gene content of 41.8%. This was not a mere consequence of our mapping strategy that precluded analysis of ambiguous events, since fusion junctions within repetitive DNA, fragile sites and regions of low gene density were also reported and validated (Supplemental Fig. 4B,C). All genes harboring fusion junctions in these data sets are provided in Supplemental Table 3A, and ontology searches revealing disruption of genes implicated in critical cellular functions are detailed in Supplemental Table 3B. Although no definitive inter-sample variation in ontological associations is present, the enrichment of genes involved in cell division and chromosome partitioning disrupted in *LIG4*^−/−^ and *LIG3*^−/−:*NC3*^ samples with potentially enhanced A-NHEJ function remained statistically significant after multiple testing correction (*P* = 0.017). This is intriguing given the propensity of these cells for escape from telomere-driven crisis and cellular transformation via telomerase reactivation (Jones et al. 2014). Conjointly, we sequence-verified fusion junctions within the *HMGA2* (Li et al. 2011) gene (Chr12q14.3) and Chr3p21.31 region implicated in the regulation of telomerase activity (Fig. 3C; Cuthbert et al. 1999) from *LIG3*^−/−^ and *PARP1*^−/−^ samples. Such gene-disrupting recombinations might facilitate the relief from active repression of telomerase function and secure cellular immortalization. Gene Set Enrichment Analysis (GSEA) established an enrichment in genes containing binding motifs for Wnt pathway (LEF-1 *P* = 9.35 × 10^−12^, TCF3 *P* = 5.12 × 10^−7^), SP1 (*P* = 2.2 × 10^−8^), and NFAT (*P* = 2.82 × 10^−9^) transcription factor components of *TERT* gene regulatory networks (Supplemental Table 3C; Kyo et al. 2000; Chebel et al. 2009; Hoffmeyer et al. 2012), revealing further potential mechanisms by which distal chromosomal locations could be juxtaposed and recombined with consequences for telomere length stabilization and malignant transformation.

Repetitive DNA sequences (Cooper et al. 2011), including fragile sites (Minca and Kowalski 2011; Thys et al. 2015) and *Alu* (Gu et al. 2015) have well-documented associations with genome instability via replication fork-stalling (Ozeri-Galai et al. 2011) and stimulation of homology-based recombination processes (Mizuno et al. 2013). Although we cannot determine the initiating DNA breakpoint for the telomere fusion events we have sequenced, we did not find any significant coincidence of inter-chromosomal fusion junctions with different classes of DNA repeats or fragile sites (Supplemental Fig. 4B,C), indicating that repetitive DNA content alone does not confer a predisposition for the ultimate fusion ligation. We next investigated junction-proximal sequence context and discovered a unique and significant enhancement (sixfold over WT; *P* ≤ 0.001) in the incidence of non-B DNA structures (Cer et al. 2013) within 500 bp of *LIG3*^−/−^:*LIG4*^−/−^ inter-chromosomal fusion junctions (Supplemental Fig. 4D). Mean GC content (Costantini et al. 2006; Pratto et al. 2014) of junction-proximal sequence extracted for these and *LIG4*^−/−^ samples (Supplemental Fig. 4E) was also correspondingly lower than WT cells (*P* = 0.0477 and *P* = 0.0445 in respective pairwise analyses), supporting the potential for distinct mechanisms and for sequence context to be permissive for long-range LIG4-independent repair. Through comparison of all MRC5^*HPVE6E7*^ and HCT116 inter-chromosomal fusion junctions with randomly generated genomic positions, we uncovered a significant 1.6-fold reduction in frequencies of fusion junctions (*P* ≤ 0.001) within 100 bp of homopolynucleotide (Ma et al. 2012) runs (Supplemental Fig. 4F,G) that may indicate these features are largely refractory or inaccessible to repair enzymes (Cohanim and Haran 2009).

### Intra-chromosomal fusions exhibit asymmetrical processing

**Figure 4.**
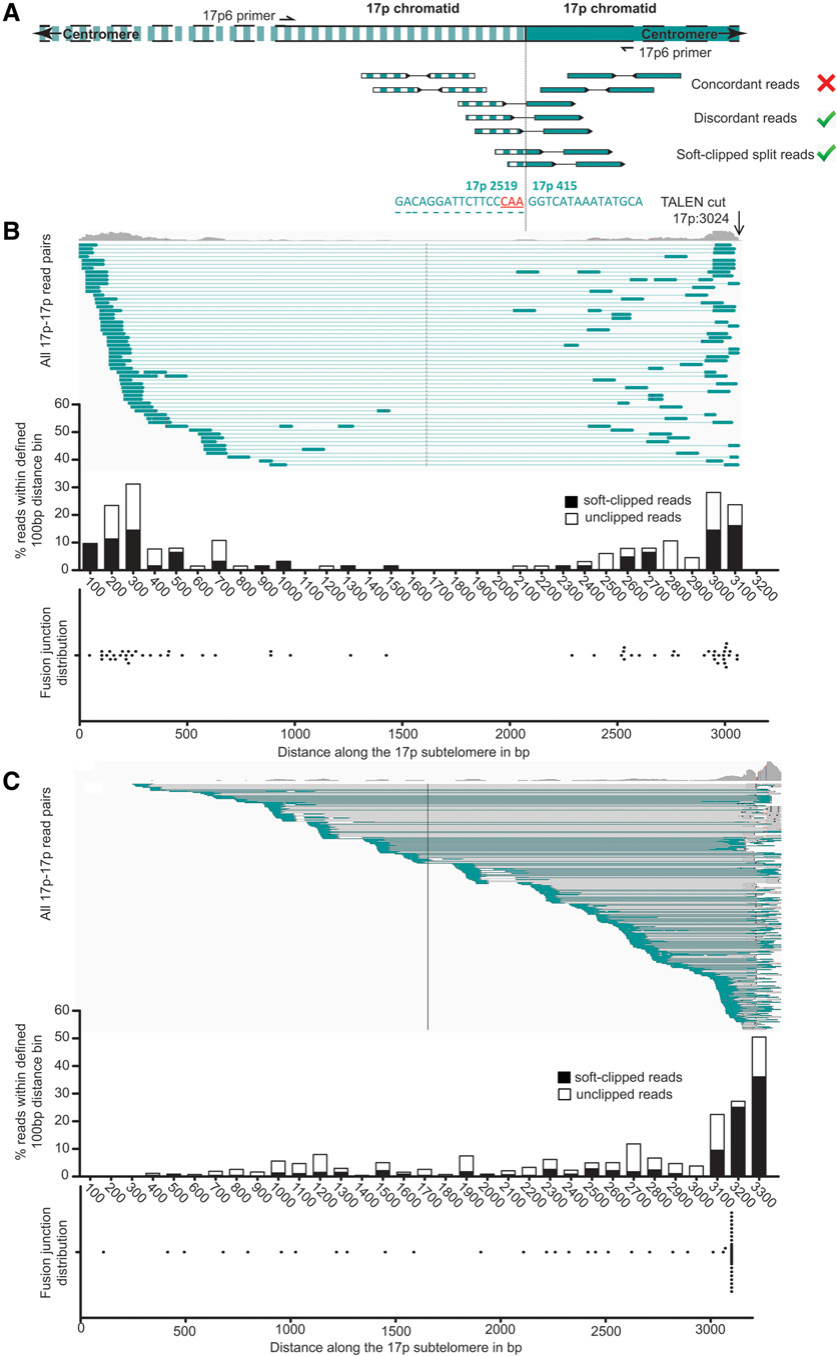
Characteristics of 17p telomere intra-chromosomal fusions sequenced from MRC5^*HPVE6E7*^ cells undergoing telomere-driven crisis or HCT116 cells undergoing 17p TALEN-induced DNA damage and repair. (*A*) Cartoon representation of a head-to-head intra-chromosomal fusion between two 17p chromatid telomeres (one striped and one blocked color) identified using discordant and soft-clipped read pairs generated by Illumina HiSeq 2000 paired-end sequencing of fusion amplicons from 17p TALEN-transfected HCT116 cells. Asymmetric subtelomeric distributions of sequencing reads mapping 17p intra-chromosomal fusions in t48 HCT116 WT (*B*) and crisis-stage MRC5^*HPVE6E7*^ cells (*C*). The *top* panel of each series displays the read frequency distribution and individual read pair linkages along the 17p subtelomere visualized using IGV software (Farré et al. 2015) (centromere to telomere orientation illustrated *left* to *right*). The 17p TALEN cleavage site 3024 bp telomeric of the 17p6 fusion primer is indicated with an arrow. Two conspicuous frequency peaks in the HCT116 sample map the long (>2 kb) and short (<1 kb) 17p chromatids that comprise the characteristic asymmetric intra-chromosomal fusions observed for all HCT116 samples. Asymmetry in the MRC5^*HPVE6E7*^ intra-chromosomal fusions results almost exclusively from the fusion of one chromatid containing telomere repeats (IGV peak ≥3050 bp from 17p6) and one resected into 17p subtelomeric sequence. The *central* panel contains a frequency plot (with 100-bp binning) of full-length unclipped (white) and soft-clipped (black) discordant read pairs used to map precise individual fusion junction positions (*lower* panel) along the 17p subtelomere.

We have proposed that the capacity of cells to escape from a telomere-driven crisis may depend on the relative balance between short-range localized intra-chromosomal recombination and more extensive LIG4-dependent inter-chromosomal interactions incompatible with proper chromosomal segregation and mitosis (Jones et al. 2014). We considered that different processes might operate to catalyze inter- compared with intra-chromosomal fusions. We sought to address this issue by determining whether the frequency or characteristics of 17p intra-chromosomal fusion events were distinct from inter-chromosomal events and if they could be affected by ablation of *LIG3* and *LIG4* that function in A-NHEJ and C-NHEJ pathways, respectively. To examine the mutational characteristics of intra-chromosomal fusion events, we filtered discordant pairs of sequence reads mapping only to the 17p subtelomere based on orientation to evaluate head-to-head fusion events (Fig. 4A) for each 17p TALEN-transfected HCT116 (Fig. 4B; Supplemental Fig. 5) and crisis-stage MRC5^*HPVE6E7*^ sample (Fig. 4C). Mapping the subtelomeric distributions of sequence reads and fusion junctions revealed a striking asymmetry inherent to all samples. Junction sequence authentication confirmed a predominance of heterogeneous fusions between chromatids of disparate lengths (Supplemental Table 4). Asymmetric resection from the start of the 17p telomere repeat arrays was also determined for the MRC5^*HPVE6E7*^ intra-chromosomal fusions, indicating that the effect is independent of the initial focal point or nature of telomere insult and not peculiar to telomerase-expressing cell lines (Fig. 4C). We used comparative mapping to the informative XpYp telomere-adjacent haplotypes in MRC5 cells (Supplemental Fig. 1B; Capper et al. 2007) to establish the involvement of a single allele, consistent with sister chromatid fusion.

As anticipated, 98%–100% of fused chromatids in the HCT116 cells did not extend beyond the TALEN cleavage site at 17p position 3024 (Supplemental Table 4), although a subset of junctions most prevalent in the *LIG3*^−/−:*NC3*^ samples (13.89% of chromatids) were located within the 17p telomere repeats and may represent fusions that have occurred independently of, or following, faithful repair of the TALEN-induced DSB. Such events were notably absent from all junctions sequenced from the *LIG3*^−/−^:*LIG4*^−/−^ cells. Although sparsely populated with fusion junctions, the central 2 kb of the 17p subtelomere was not refractory to fusion amplification, or sequencing, since we were able to map MRC5^*HPVE6E7*^ and some HCT116 events to this location, particularly at the later experimental time point (Supplemental Fig. 5). Additionally, we detected a group of fusion junctions within 1.0–1.2 kb of the TALEN target site isolated from the t48 *PARP1*^−/−^ and *LIG3*^−/−^:*LIG4*^−/−^ cells. Taken together, these results demonstrate a distinct asymmetry in the processing of sister chromatids prior to intra-chromosomal telomere fusion.

### Inter- and intra-chromosomal telomere fusions are differentially processed and ligated

In contrast to the inter-chromosomal fusions that exhibited clear inter-sample variability in frequency and sequence characteristics, we discovered a conspicuous homogeneity among intra-chromosomal fusions associated with all samples, irrespective of their genetic background (Supplemental Fig. 2G). These fusions were most abundant in the *TP53*^−/−^ samples, in line with their raised inter-chromosomal fusion frequencies (Supplemental Fig. 1C) and further evidencing the significant genomic instability resulting from the loss of this mitotic checkpoint and subtelomeric protection (Tutton et al. 2015). The absence of functional LIG3 or LIG4 did not significantly reduce the incidence of 17p intra-chromosomal fusion events detected, and importantly, the intra-chromosomal fusion frequency in the *LIG3*^−/−^:*LIG4*^−/−^ samples was not notably diminished by the dual compromise of C- and A-NHEJ repair, suggesting sufficient redundancy in the pathways to maintain intra-chromosomal ligation activity. This result distinguishes intra-chromosomal from inter-chromosomal fusions that demonstrate greater dependency on LIG4 (Ghezraoui et al. 2014).

**Figure 5.**
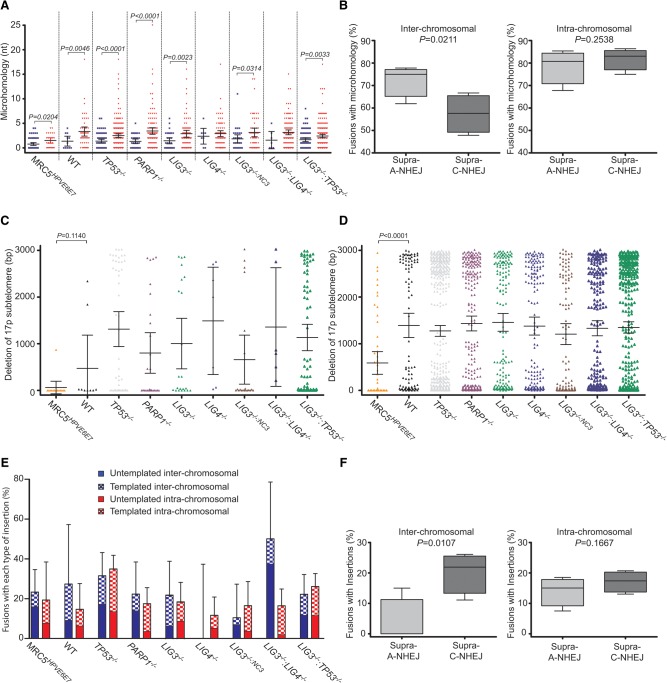
Differential processing of inter- and intra-chromosomal fusion junctions in crisis-stage MRC5^*HPVE6E7*^ and 17p TALEN-treated HCT116 cell lines. (*A*) Microhomology usage (≥1 bp sequence overlap) at each fusion junction plotted as a scatter chart with mean values and 95% CI for each sample. Mean nucleotides of microhomology at inter-chromosomal fusion junctions (blue crosses) are significantly lower (two-tailed unpaired *t*-test, Welch's correction) for all samples (except *LIG4*^−/−^ and *LIG3*^−/−^:*LIG4*^−/−^) than the corresponding intra-chromosomal fusion junctions (red triangles). (*B*) The approximate 25% increase in percentages of inter-chromosomal (*left*) but not intra-chromosomal (*right*) fusion junctions with microhomology identified for Supra-A-NHEJ (*LIG4*^−/−^ and *LIG3*^−/−:*NC3*^) over Supra-C-NHEJ (*PARP1*^−/−^ and *LIG3*^−/−^) samples is statistically significant (*P* = 0.0211, one-tailed unpaired *t*-test with Welch's correction). Resection from the 17p TALEN cleavage site (HCT116) or telomere repeats (MRC5^*HPVE6E7*^) for each 17p inter-chromosomal (*C*) or intra-chromosomal (*D*) fusion chromatid plotted as a scatter chart with mean base pair and 95% CI, revealing notable length asymmetry of intra-chromosomal fusion partners. The greater than twofold lower mean resection of MRC5^*HPVE6E7*^ compared with HCT116 WT chromatids was statistically significant (*P* < 0.0001) by one-tailed unpaired *t*-test with Welch's correction. (*E*) The percentages of inter-chromosomal (blue) and intra-chromosomal (red) fusion junctions containing insertions (<50 bp) plotted with 95% CI including subclassification of events as templated (≥2 nt sequence similarity; checkered boxes) or untemplated (no BLAST nucleotide alignment; solid boxes). (*F*) The statistical significance (*P* = 0.0107) of the approximate fivefold increase in mean percentage of inter-chromosomal (*left*) but not intra-chromosomal (*right*) junctions with insertions for Supra-A-NHEJ samples was evaluated by one-tailed unpaired *t*-test with Welch's correction.

To investigate the underlying mechanisms explaining altered fusion frequencies, we compared junction processing in the inter- and intra-chromosomal fusions sequenced for each sample. LIG3-mediated ligation is associated with microhomology usage, whereas the rapid kinetics of LIG4-mediated C-NHEJ generally preclude extensive processing and resection required for homology searching (Shibata et al. 2011). Consistent with our published observations (Letsolo et al. 2010; Jones et al. 2014), we found that mean microhomology usage at inter-chromosomal junctions was greater for *LIG4*^−/−^ cells (2.38 nt) than *LIG3*^−/−^ cells (1.44 nt) (Fig. 5A; Supplemental Fig. 6A). This suggests that the mechanisms of inter-chromosomal fusion in LIG4-deficient cells are more dependent on microhomology usage characteristic of A-NHEJ-mediated repair, than are fusions that are mediated by LIG4. In contrast, intra-chromosomal junctions derived from *LIG3*^−/−^ and *LIG4*^−/−^ cells displayed no difference in mean microhomology usage (2.9 nt each) (Fig. 5A; Supplemental Fig. 6B). The variable frequencies of inter-chromosomal fusion events precluded direct statistical analysis of these samples, so we clustered the samples according to their enhanced (Supra) potential for A-NHEJ (*LIG4*^−/−^ and *LIG3*^−/−:*NC3*^) or C-NHEJ (*PARP1*^−/−^ and *LIG3*^−/−^) function. This comparison revealed a significant 20% reduction (*P* = 0.0211) in the number of inter-chromosomal junctions at which microhomology usage was detected for the Supra-C-NHEJ events, but no difference in proportions at the intra-chromosomal junctions (Fig. 5B; Supplemental Fig. 6C). Combining all HCT116 samples, the mean number of nucleotides of microhomology measured was significantly higher (*P* = 0.0006) at intra-chromosomal junctions (3.01 nt) compared with inter-chromosomal junctions (1.62 nt). These disparities were also apparent in each of the individual HCT116 genetic backgrounds (with the exception of *LIG4*^−/−^ and *LIG3*^−/−^:*LIG4*^−/−^) and in junctions sequenced from crisis-stage MRC5^*HPVE6E7*^ cells (Fig. 5A; Supplemental Fig. 6D). These data lead us to infer a greater dependency on A-NHEJ repair with microhomology usage at telomeric intra-chromosomal junctions. This is supported by the persistence of intra-chromosomal fusions in the *LIG4*^−/−^ and *LIG3*^−/−^:*LIG4*^−/−^ samples, where we hypothesize that LIG1 functionally compensates for the lack of LIG3 in A-NHEJ.

We investigated differential resection (Miller et al. 2011) at inter- and intra-chromosomal fusion junctions by analyzing the distribution of fusion junctions with respect to the 17p telomeric-TALEN cut site for each sample (Fig. 5C). Asymmetrical processing of sister chromatids (Fig. 4B), was reflected in the clear bimodal distributions observed for the intra-chromosomal junctions (Fig. 5D). Although fewer inter-chromosomal fusion events could be recorded owing to the more complex spectrum of recombinations, bimodal distributions were still apparent, suggesting that 17p telomeres at fusion segregate into long or short chromatid groupings irrespective of whether the fusion occurs between a sister chromatid or distant genomic site. Although this is consistent with asymmetrical processing, it was not possible to identify the two participating telomeres for each inter-chromosomal fusion event. Resection at *LIG4*^−/−^ compared with *LIG3*^−/−^ inter-chromosomal junctions sequenced from t120 samples was substantially greater (mean of 1908 bp compared with 751 bp; *P* < 0.05) and, combined with the notable lack of any *LIG4*^−/−^ inter-chromosomal fusion junctions containing the TALEN footprint, may reflect a shift toward A-NHEJ-mediated inter-chromosomal events requiring resection to reveal microhomology in the absence of LIG4 (Oh et al. 2014). This is consistent with the increased microhomology usage described at the inter-chromosomal fusion junctions sequenced from the *LIG4*^−/−^ cells (Fig. 5A; Supplemental Fig. 6A). The 1.3-fold to fivefold enhanced incidence of the TALEN target sequence determined for all other samples’ inter-chromosomal fusion junctions compared with intra-chromosomal junctions (Supplemental Fig. 2A) is again supportive of a bias toward C-NHEJ repair at inter-, but not intra-chromosomal telomere fusions.

### Intra-chromosomal telomere fusions are informative of differential DNA polymerase activity

We sought to discover whether insertion frequency was differentially affected by DNA ligase usage at inter-chromosomal compared with intra-chromosomal fusion junctions (Fig. 5E). We were able to identify short (<50 bp) insertions at junctions sequenced from MRC5^*HPVE6E7*^ and all HCT116 cell lines that included complex events, such as localized duplications of 17p telomere sequence, as well as simple untemplated nucleotide insertions (Supplemental Fig. 7A). Insertion rates were comparable at inter- and intra-chromosomal fusion junctions for all samples except the *LIG4*^−/−^ and *LIG3*^−/−^:*LIG4*^−/−^ samples, in which inter-chromosomal insertion event frequencies were distinct from the corresponding intra-chromosomal events for these and all other samples. Whereas a deficiency for LIG4 alone resulted in low numbers of inter-chromosomal fusions (Supplemental Fig. 2G) and the absence of any junction insertions (Fig. 5E), the dual loss of LIG3 and LIG4 resulted in a considerable increase in the proportions of inter-chromosomal junctions with insertions (mean 1.8-fold above WT), and these were predominantly untemplated. As with other parameters of junction processing, this differential between C-NHEJ and A-NHEJ-mediated inter-chromosomal fusions was not conserved at the intra-chromosomal fusion junctions (Fig. 5F), where the proportions of templated events were higher than untemplated events for all samples (mean twofold higher).

We next explored the possibility that the asymmetrical processing of fusion chromatids we observed (Fig. 5C,D) might reflect the distinct DNA polymerases that replicate leading- versus lagging-strand DNA. Leading-strand DNA synthesis is coordinately mediated by DNA polymerases delta and epsilon, resulting in minimal error incorporation (Nick McElhinny et al. 2008; Lujan et al. 2012; Johnson et al. 2015), whereas lagging-strand replication occurs by ligation of Okazaki fragments synthesized by DNA polymerases alpha and delta with reduced proof-reading capacity (Stith et al. 2008; Reijns et al. 2015). We hypothesized that the shorter chromatid partner of each 17p intra-chromosomal fusion might result predominantly from lagging-strand DNA synthesis and thus display a higher frequency of nonconstitutive nucleotide changes (Supplemental Fig. 7B). We segregated sequence read pairs into their short (centromeric) and long (telomeric) components and determined a clear trend (*P* = 0.0524) toward higher error incorporation into the centromeric reads that may implicate DNA polymerase alpha (Supplemental Fig. 7C). This differential error rate was statistically significant (*P* = 0.0126) when the intriguingly antithetical *LIG3*^−/−^ sample was excluded from the comparison (Supplemental Fig. 7D). These results provide tentative credence to a model of intra-chromosomal fusion mediated by compound ligation of differentially replicated DNA strands.

## Discussion

We have previously demonstrated a functional requirement for LIG3 in expediting cellular escape from telomere-driven crisis (Jones et al. 2014). Here, we provide new insight into the underlying mechanisms involved, addressing formerly intractable scientific questions concerning the balance of DNA repair processes involved in inter- and intra-chromosomal recombinations. Through a rigorous and systematic examination of telomere recombinations in genetic models selectively compromised for constituents of C- and A-NHEJ, we have been able to distinguish the exclusive contributions of LIG3 and LIG4 and provide evidence for the functional involvement of LIG1 for the first time in human cells. We demonstrate a severe deficiency in the long-range inter-chromosomal fusion capacity of cells lacking LIG4, but reveal an unexpected proficiency for intra-chromosomal sister chromatid events in cells lacking both LIG3 and LIG4.

By high-resolution mapping of more than 400 unique fusion events between specific telomeres and nontelomeric loci, we have revealed a remarkable genome-wide dispersal of recombinations, with a disproportionate clustering on the experimentally targeted chromosome. These data demonstrate the enormous scale of genomic instability that can arise from a single chromosomal subtelomeric DSB or short dysfunctional telomere. This observation is of particular significance as stochastic telomeric deletion is detected in normal human cells (Baird et al. 2003) and such telomeres are capable of fusion (Capper et al. 2007). We identified complex inter-chromosomal fusion events incorporating multiple remote genomic loci, implicating propagation of DNA damage and replication stress from the original subtelomeric insult to elicit fusogenic DSBs throughout the genome. We determined a significant enrichment of inter-chromosomal fusion junctions within coding sequence with anticipated deleterious consequences that may explain the failure of cells to escape a telomere-driven crisis in the absence of LIG3 when inter-chromosomal events are proportionally more abundant (Jones et al. 2014). The parity of fusion spectra between the crisis-stage MCR5^*HPVE6E7*^ sample with global telomere dysfunction and the diverse HCT116 cell lines with a single chromosome targeted telomere destabilization signifies a common basis to this coding sequence association, rather than a cell line or DNA repair pathway-specific effect (Supek and Lehner 2015). Thus, our data clearly show that dysfunction at a single telomere is highly mutagenic, resulting in large-scale genomic rearrangements with the potential to drive clonal evolution.

The high frequency of fusion junctions within genes suggests that replication timing and chromatin structure may affect the fusogenic potential of genomic loci. Gene-rich regions are archetypically early replicating euchromatin associated with low mutational burden (Stamatoyannopoulos et al. 2009). In a model of DNA replication-coupled repair, however, these loci represent the most likely sources of copy number variation, increasing local substrate availability at locations where repair and replication enzymes are already colocalized. As such, these loci may also present enhanced opportunity for fusion by replication-independent NHEJ as a means of rapid DSB repair, resulting in mitotic arrest (Hayashi et al. 2015). Cell cycle regulation of template availability and processing may also affect the mechanism of telomere fusions (Escribano-Díaz et al. 2013). We were able to sequence rare telomere-genomic fusions from *LIG3*^−/−^:*LIG4*^−/−^ cells, reinforcing the notion of the coincidence of fusion foci with DNA replication, since LIG1 is the most rational protagonist mediating these LIG4-independent inter-chromosomal events (Arakawa et al. 2012; Lu et al. 2016). Further support arises from our finding of a conspicuous and significant association of *LIG3*^−/−^:*LIG4*^−/−^ junction-proximal sequence with non-B DNA structures (Cooper et al. 2011; Cer et al. 2013) and a trend toward increased coincidence with fragile sites for *LIG4*^−/−^ junctions, implicating replication fork-stalling (Vissers et al. 2009; Minca and Kowalski 2011; Ozeri-Galai et al. 2011; Mizuno et al. 2013) as a determinant of chromosome breakage and/or fusion. The A-NHEJ DNA polymerase theta also functions at the earliest stages of DNA replication and may, therefore, play a role in the introduction of the residual templated insertions resolved in these LIG4-deficient cells (Fernandez-Vidal et al. 2014).

Our data also support a complex interplay between DNA replication and repair at the telomere. Intra-chromosomal fusion frequencies measured for *LIG3*^−/−^ and *LIG4*^−/−^ samples were similar, and surprisingly, were exceeded by the *LIG3*^−/−^:*LIG4*^−/−^ samples, revealing that LIG1 is sufficient to catalyze these fusion events in addition to its vital role as a replicative ligase. The pronounced asymmetry of telomeric fusions is suggestive of replication, rather than resection, imbalances between the fusing partners. We were able to uncover a skew in error-incorporation segregating with short versus long paired chromatids that may reflect the ligation of leading with lagging-strand DNA. The reduction in proofreading capacity of lagging-strand polymerases (Reijns et al. 2015), coupled with increased fork stalling at telomeric locations (Lormand et al. 2013) could result in an incompletely replicated template (Chow et al. 2012) that is ligated with the leading strand full-length chromatid to create a nonpalindromic fusion (Stohr et al. 2010). The higher incidence of templated insertions we detected at intra-chromosomal junctions also suggests an active contribution of DNA synthesis to repair (Lowden et al. 2011), conceivably involving the A-NHEJ-associated DNA polymerase theta (Yousefzadeh et al. 2014; Ceccaldi et al. 2015; Mateos-Gomez et al. 2015) or alpha (Reijns et al. 2015) by way of incomplete primer removal. Thus, the more prominent role of LIG1 in intra-chromosomal fusion events provides a mechanistic link to the asymmetric architecture via distinctive chromatid replication.

Using cell lines selectively compromised in different components of NHEJ repair, we have identified a crucial role for LIG4 in mediating long-range inter-chromosomal fusions between damaged telomeres and diverse genomic locations. Consistent with the hallmarks of C-NHEJ (Ghezraoui et al. 2014; Oh et al. 2014), these telomere-genomic interactions showed reduced microhomology usage at junctions in comparison with intra-chromosomal telomere fusion events. Although the frequency of telomere-genomic events was severely reduced in cells lacking LIG4, there was no analogous impact on intra-chromosomal fusion events, resulting in a notable homogeneity of parameters examined among all samples. We determined an increased incidence of microhomology usage and templated insertions at intra-chromosomal telomere fusion junctions, indicating that TALEN-induced subtelomeric DSBs initiate A-NHEJ repair activity similar to that resulting from the fusion of short-dysfunctional telomeres (Capper et al. 2007; Rai et al. 2010). Templated insertions observed at inter-chromosomal fusions may result from synthesis by polymerase mu across a discontinuous template, stabilized by LIG4 in concert with Ku and X-ray repair cross-complementing protein 4 (XRCC4) (Nick McElhinny et al. 2005). These insertions were proportionally increased (relative to untemplated insertions) in *LIG3*^−/−^ and WT samples and coordinately reduced in *LIG3*^−/−:*NC3*^, *LIG4*^−/−^, and *LIG3*^−/−^:*LIG4*^−/−^ samples. Our data therefore demonstrate a predominant role for LIG4-dependent C-NHEJ in mediating inter-chromosomal telomere fusion, with LIG1/3-dependent A-NHEJ prevailing in the fusion of sister chromatids displaying short dysfunctional telomeres.

## Methods

### Cells

The wild-type (WT) HCT116 diploid human colorectal carcinoma cell line was used along with seven genetically engineered cell lines (Bunz et al. 1998; Jones et al. 2014; Oh et al. 2014): (1) *TP53*^−/−^; (2) *PARP1*^−/−^; (3) *DNA ligase 3*^−/−^ complemented with mitochondrial DNA ligase 3 (to render it viable): *LIG3*^−/−^; (4) *DNA ligase 4*^−/−^: *LIG4*^−/−^; (5) *DNA ligase 3*^−/−^ complemented with mitochondrial and nuclear ligase 3: *LIG3*^−/−:*NC3*^; (6) *DNA ligase 3* and *4* combined knockouts: *LIG3*^−/−^:*LIG4*^−/−^; (7) *DNA ligase 3* and *TP53* combined knockouts: *LIG3*^−/−^:*TP53*^−/−^. They were generated using recombinant adenoviral-associated (rAAV) and CRISPR/Cas9 (*LIG3*^−/−^:*LIG4*^−/−^ line only) targeting methods, as described (Khan et al. 2011; Oh et al. 2014; B Ruis, T Takasugi, S Oh, EA Hendrickson, unpubl.). These cell lines represent our models of DNA repair pathways that are selectively compromised. Cells lacking TP53 have defective cell cycle checkpoints (Hartwell 1992; Bunz et al. 1998; Davoli et al. 2010). Cells deficient for PARP1 or LIG3 are compromised for alternative NHEJ (A-NHEJ) repair, whereas cells deficient in LIG4 fail to execute classical NHEJ (C-NHEJ) repair. The HCT116 *LIG3*^−/−^ line used here is sufficient for mitochondrial LIG3 but deficient in the nuclear LIG3 that is functional in genomic NHEJ (Oh et al. 2014). The HCT116 *LIG3*^−/−:*NC3*^ can be considered a model of supraphysiological nuclear LIG3 expression. The *LIG3*^−/−^:*LIG4*^−/−^ double knockout cells are compromised in both A-NHEJ and C-NHEJ repair.

### 17p subtelomere TAL effector nucleases

TAL Effector Nucleotide Targeter 2.0 software (Cornell University) (Doyle et al. 2012) was used to design two separate TAL Effector Nuclease (TALEN) pairs to target a unique telomere-adjacent human 17p subtelomeric sequence. TALEN pairs and surrogate reporters for targeting validation were custom synthesized by Labomics S.A., and endotoxin-free transfection-quality plasmid preparations were purified from Stbl3 competent cells (Invitrogen) using Nucleobind Xtra Midi Plus kits (Macherey-Nagel). Plasmid identity was confirmed by XbaI restriction enzyme mapping and Sanger DNA sequencing. TALEN target site cleavage activity was detected by 17p sequence-specific nested PCR using 300 pg initial input DNA, followed by Sanger DNA sequencing of mutated sites and reamplified 17p fusion molecules. The 17p TALEN pair resulting in the highest frequency of 17p telomere fusions (3.6 × 10^−4^ versus 1.6 × 10^−4^/diploid genome) was selected for use in all experiments analyzed by Illumina paired-end sequencing. There was no differential impact on cell viability associated with these TALEN pairs. The use of either TALEN pair reduced HCT116 cell viability from the 75%–80%, typically seen 48 h after transfection with no DNA or empty vector control, to 50% viability, as assessed by microscopy.

### Telomere fusion PCR

Telomere fusion amplicons were generated from sample input genomic DNA (50 ng from HCT116 or 12.5 ng from the MRC5^*HPVE6E7*^ cell line) by multiplex long-range PCR using primers targeting the 17p, XpYp, and 21q family of homologous telomeres (17p6, XpYpM, and 21q1 primers, respectively) (Britt-Compton et al. 2006; Letsolo et al. 2010). This resulted in mixed pools of fusion amplicons with divergent molecular weights. For standard experiments, 6–12 individual replicate reactions were typically resolved by 0.5% TAE agarose gel electrophoresis for detection on Southern blots using radiolabeled telomere-adjacent probes specific for each telomere end. To estimate telomere fusion frequency in each sample, the number of resolved nonconstitutive fusion amplicons revealed by Southern blotting was summarized and divided by the number of input DNA molecules calculated in diploid genomes (1 diploid human genome approximates 6 pg DNA). Thus, fusion frequencies are expressed as events per diploid genome.

### Illumina HiSeq 2000 paired-end sequencing

For Illumina HiSeq 2000 paired-end sequencing, (1) 85 telomere fusion PCR replicates were prepared from HCT116 t48 DNA samples; (2) 170 replicates from HCT116 t120 DNA samples; and (3) 192 replicates from the crisis-stage MRC5^HPVE6E7^ sample. Random PCR wells were selected for cross-checking validation prior to the replicates for each sample being pooled and post-PCR purified using Agencourt AMPure XP magnetic beads with elution in nuclease-free water. Aliquots of each sample were taken pre- and post-purification for a comparative assessment and to check purification efficiency. Sequencing of the MRC5^*HPVE6E7*^ fusion amplicons was conducted by BGI Tech. Yields approached 10 Mb of data, consisting of 60 million read pairs. Sequencing of the HCT116 TALEN-treated samples was performed in collaboration with the Oxford Genomics Centre using the Nextera XT sample prep kit. Read yields were in the range of 40 to 75 million per sample, corresponding to 10–15 Mb of data per sample. Details are contained in Supplemental Table 2A.

### Identifying telomere fusion events

All custom scripts used to map and analyze these data sets are available as Supplemental Scripts or can be downloaded from GitHub (https://github.com/nestornotabilis/GenomeResearch_2016_scripts). The associated Java code can be downloaded (https://github.com/nestornotabilis/WGP-Toolkit).

Inter-chromosomal fusion events were identified from those discordant sequence read pairs that mapped to both a custom subtelomeric reference (comprised of 17p, XpYp, and the 21q family subtelomeric sequences appended with all known telomere variant repeats) (Jones et al. 2014) and a modified human genome hg19 reference (supplemented with updated subtelomeric sequences and the 17p, XpYp, and 21q sequences) (Stong et al. 2014).

Subtelomeric intra-chromosomal fusion events were identified from those discordant read pairs that mapped to a single subtelomeric reference in the same orientation. All read pairs were subsequently filtered on a MAPQ (mapping quality) value ≥0 to exclude ambiguous (mapping more than one location) or poor quality mappings and subjected to iterative high-resolution interrogation using BLAST to ensure unique and accurate mapping.

## Data access

BAM files containing trimmed and filtered data for each MRC5^*HPVE6E7*^ and HCT116 sample have been submitted to EMBL-EBI ArrayExpress (http://www.ebi.ac.uk/arrayexpress) under accession number E-MTAB-3811.
